# Implementation and Impact of Intimate Partner Violence Screening Expansion in the Veterans Health Administration: Protocol for a Mixed Methods Evaluation

**DOI:** 10.2196/59918

**Published:** 2024-08-28

**Authors:** Galina A Portnoy, Mark R Relyea, Melissa E Dichter, Katherine M Iverson, Candice Presseau, Cynthia A Brandt, Melissa Skanderson, LeAnn E Bruce, Steve Martino

**Affiliations:** 1 VA Connecticut Healthcare System West Haven, CT United States; 2 Yale School of Medicine New Haven, CT United States; 3 VA Center for Health Equity Research and Promotion (CHERP) Corporal Michael J. Crescenz VA Medical Center Philadelphia, PA United States; 4 School of Social Work Temple University Philadelphia, PA United States; 5 Women’s Health Sciences Division of the National Center for PTSD VA Boston Healthcare System Boston, MA United States; 6 Department of Psychiatry Boston University School of Medicine Boston, MA United States; 7 Intimate Partner Violence Assistance Program, Care Management and Social Work Service Veterans Health Administration Washington, DC United States

**Keywords:** screening, intimate partner violence, implementation, evaluation, national rollout, health care, quality improvement, veterans

## Abstract

**Background:**

Intimate partner violence (IPV) is a significant public health problem with far-reaching consequences. The health care system plays an integral role in the detection of and response to IPV. Historically, the majority of IPV screening initiatives have targeted women of reproductive age, with little known about men’s IPV screening experiences or the impact of screening on men’s health care. The Veterans Health Administration (VHA) has called for an expansion of IPV screening, providing a unique opportunity for a large-scale evaluation of IPV screening and response across all patient populations.

**Objective:**

In this protocol paper, we describe the recently funded Partnered Evaluation of Relationship Health Innovations and Services through Mixed Methods (PRISM) initiative, aiming to evaluate the implementation and impact of the VHA’s IPV screening and response expansion, with a particular focus on identifying potential gender differences.

**Methods:**

The PRISM Initiative is guided by the Reach, Effectiveness, Adoption, Implementation, and Maintenance (RE-AIM) and Consolidated Framework for Implementation Research (CFIR 2.0) frameworks. We will use mixed methods data from 139 VHA facilities to evaluate the IPV screening expansion, including electronic health record data and qualitative interviews with patients, clinicians, and national IPV program leadership. Quantitative data will be analyzed using a longitudinal observational design with repeated measurement periods at baseline (T0), year 1 (T1), and year 2 (T2). Qualitative interviews will focus on identifying multilevel factors, including potential implementation barriers and facilitators critical to IPV screening and response expansion, and examining the impact of screening on patients and clinicians.

**Results:**

The PRISM initiative was funded in October 2023. We have developed the qualitative interview guides, obtained institutional review board approval, extracted quantitative data for baseline analyses, and began recruitment for qualitative interviews. Reports of progress and results will be made available to evaluation partners and funders through quarterly and end-of-year reports. All data collection and analyses across time points are expected to be completed in June 2026.

**Conclusions:**

Findings from this mixed methods evaluation will provide a comprehensive understanding of IPV screening expansion at the VHA, including the implementation and impact of screening and the scope of IPV detected in the VHA patient population. Moreover, data generated by this initiative have critical policy and clinical practice implications in a national health care system.

**International Registered Report Identifier (IRRID):**

PRR1-10.2196/59918

## Introduction

### Overview

Intimate partner violence (IPV), including physical, sexual, and psychological aggression, is a significant public health problem with far-reaching consequences. Experiencing IPV is associated with serious negative physical and psychological outcomes among civilians and veterans alike [1,2]. Research shows that women veterans are at increased risk of experiencing violence in relationships compared to civilian women [1]. Moreover, IPV is common, yet critically understudied, among veteran men.

Reported rates of IPV experience vary widely across studies, largely due to methodological differences [3,4]. Although US women veterans are more likely to experience lifetime IPV than men (approximately 45% vs 36%), the prevalence of past-year IPV is similar, at approximately 30% [5]. Research has found that as many as 55% of women veterans experience IPV in their lifetimes [6]. Women’s experiences of IPV are associated with adverse physical and mental health, including cardiovascular and respiratory problems, chronic pain, reproductive health challenges, posttraumatic stress, anxiety, depression, substance use, and elevated risk for suicide [2,7-10]. For veteran men, IPV experience is associated with poorer overall mental health; greater occupational impairment; and higher rates of depression, smoking, and heavy and binge drinking [11,12]. Despite high rates of IPV experience and adverse outcomes for men, little research has examined IPV screening and referral outcomes for this population. Moreover, although evidence demonstrates that transgender and nonbinary patients are at increased risk of IPV compared to cisgender patients [13,14], very little research has examined IPV screening and referral outcomes within this population [15].

The health care system plays an integral role in the detection of and response to IPV [16,17]. As such, the significant impact that IPV has on veterans across genders underscores the critical need for a comprehensive and effective health care response for patients seeking services through the Veterans Health Administration (VHA). In 2014, the VHA developed the national IPV Assistance Program to oversee and implement integrated services aimed at reducing the risk for IPV, including establishing IPV Assistance Program Coordinators at each Department of Veterans Affairs (VA) medical center across the country and providing clinical services and resources for IPV-related concerns through prevention, detection, and treatment [18]. Since the IPV Assistance Program’s inception, the implementation of IPV screening and response among women veterans has been an important priority area for the program.

The VHA policy for IPV detection and response parallels and expands on recommendations put forth by the US Preventive Services Task Force [19]. VHA policy requires annual IPV screening for women of reproductive age, as well as other patients who belong to a recognized high-risk group (eg, patients who are homeless or underhoused, those with co-occurring disorders, and patients with disability [20]) and provision of support, resources, and referrals [20]. Accordingly, enhancing the implementation of IPV screening and response among the women veteran patient population has been a major priority of VHA clinical practice and research efforts over the last decade. A robust body of literature has shown that IPV screening of women veterans during health care visits is essential as patients are unlikely to spontaneously disclose IPV but will often report their IPV experiences when asked by a clinician in a sensitive manner [21]. Evidence also demonstrates that screening increases the identification of IPV and enhances women veterans’ connections to and satisfaction with care, and women veterans report perceiving IPV screening as supportive, validating, and helpful [21-23].

However, there remain many system-level and clinician-reported barriers to screening women veterans, such as limited time and resources, discomfort in addressing IPV, lack of training, and competing priorities during health care visits [24,25]. Moreover, because the majority of IPV research and health care screening initiatives to date have targeted women, little is known about screening men for IPV, including men’s perceptions and experiences of screening, their willingness to disclose IPV during screening, and clinicians’ experiences and attitudes about screening men. These limitations highlight the need for a large-scale evaluation of IPV screening reach and effectiveness with men and patients of all gender identities to inform strategies for optimizing screening implementation across patient populations. To date, IPV screening implementation across health care systems, including the VHA, has targeted women of reproductive age and largely has occurred in primary care, obstetrics or gynecology, and urgent care settings. The recent VHA IPV screening expansion provides a unique opportunity for evaluation of the IPV screening and response protocol across VHA patient populations.

### Expansion of IPV Screening

In response to growing evidence demonstrating that all VHA patient populations are at risk for experiencing IPV and associated negative health consequences (eg, men [12,26], women above reproductive age [27], and transgender and nonbinary veterans [13,14]), the VA National IPV Assistance Program has called for expanded IPV screening through the implementation of a “no wrong door” approach using the Relationship Health and Safety Clinical Reminder version 3 (RHS 3.0). This approach enables a patient-centered solution for detecting IPV such that patients are screened for IPV regardless of where they receive care within the health care system. The RHS 3.0 is a 2-part IPV screener, including a 5-item primary screen for IPV [28-30] and, if triggered, a 3-item secondary screen for risk of severe and potentially lethal IPV [31].

The RHS 3.0 was developed as a clinical reminder with a note template and approved for VHA enterprise-wide installation in August 2023. The clinical reminder prompts clinicians to screen women of reproductive age at least annually and is available and recommended for use with all patients outside of this target demographic, as well. Veterans can be screened for IPV and connected with support services wherever they present for care in the health care system, resulting in a “no wrong door” approach. The RHS 3.0 is administered using a standardized template in the electronic health record (EHR). Population health implementation support tools, such as clinical reminders and note templates, leverage standardization of the EHR to help systematize screening administration and data collection across a large patient population [32]. As the largest integrated health care system in the United States, the VHA serves over 9 million patients across 172 health care facilities [33], underscoring the importance of leveraging tools available in the EHR for reaching all patients who come into contact with the health care system.

To conduct IPV screening and complete the necessary clinical reminder steps, clinicians require training through an internet-based training module available to all VHA staff or through receiving training conducted by an IPV Assistance Program Coordinator. To support the adoption of the RHS 3.0 screening expansion, the IPV Assistance Program is combining a top-down approach, which includes disseminating education and materials throughout the VHA (eg, national live and recorded trainings, an IPV screening and response toolkit, and internet-based training modules), with facility-level implementation strategies at the discretion of local IPV Assistance Program Coordinators at each medical center.

A national screening expansion of this scale requires rigorous, systematic evaluation. Although prior work exists evaluating IPV screening implementation for women veterans in primary care settings [23-25,34-37], no effort to date has systematically evaluated the implementation of IPV screening among all patients across the entire health care system. Data are needed to assess the implementation of the screening expansion, as well as the impact of screening new patient populations. In this protocol paper, we describe the recently funded Partnered Evaluation of Relationship Health Innovations and Services through Mixed Methods (PRISM) initiative. The aims of this work are to (1) evaluate the implementation of the RHS 3.0 IPV screening and response to national expansion and (2) identify the impact of IPV screening and potential gender differences. Specifically, we will assess implementation outcomes across Reach, Effectiveness, Adoption, Implementation, and Maintenance (RE-AIM) domains and examine potential differences in outcomes by patient characteristics (eg, gender, age, race, ethnicity, sexual orientation, and marital status). To examine the “no wrong door” approach, we will also identify clinical settings most and least likely to adopt IPV screening, as well as yield disclosures during screening. We will examine the impact and potential gender differences by assessing service use and clinical outcomes following positive screens and exploring experiences and perceptions of clinicians who screened and patients who disclosed IPV during screening encounters.

## Methods

### Conceptual Framework

The PRISM initiative is guided by 2 robust implementation science frameworks—the RE-AIM (outcomes framework [38]) and the updated Consolidated Framework for Implementation Research (CFIR 2.0; determinants framework [39]). Although RE-AIM helps guide the organization of evaluation outcomes, it does not necessarily explain the conditions that influence variation in outcomes across the system, including among patient subgroups and clinical settings. The CFIR 2.0 is particularly helpful for structuring the exploration of contextual factors essential for the implementation of innovative programs, including at the VHA [40], making it ideally suited to guide the evaluation of multilevel factors that impact the implementation success of the VHA IPV screening and response national expansion. Integrating these 2 frameworks will support the assessment of implementation outcomes alongside understanding potential barriers and enablers of screening implementation, clinicians’ experiences with IPV screening and patients’ experiences with being screened (particularly among newly targeted patient populations, like men), and the implementation process overall.

### Data Sources

Evaluation of the RHS 3.0 IPV screening expansion will include mixed methods data across VA health care facilities nationally. We will integrate quantitative and qualitative data sources, including EHR data and qualitative interviews (see Table 1 for a summary of data sources and outcomes). Quantitative data will be extracted from the VA’s Corporate Data Warehouse (CDW), a centralized data repository that aggregates clinical, administrative, and financial data from the VA EHR across all 139 VA medical centers and satellite clinics [41]. Our evaluation sample will include all VHA veteran patients with at least 1 outpatient VHA health care encounter during the evaluation observation period. Qualitative data sources will include semistructured interviews with veterans, clinicians, and VA leadership from the national IPV Assistance Program. The development of the semistructured interviews was guided by the CFIR 2.0 domains. Specifically, clinician interviews will focus on CFIR 2.0 constructs related to the RHS 3.0 screening and response protocol itself, the outer and inner settings (ie, their clinics and facilities), responders’ experiences and perceptions of the screening and response (including perceived impacts for patients), and the implementation process. Veteran interviews will also focus on their perceptions of being screened, including experiences with the screening process itself and resulting outcomes (eg, services offered or received).

**Table 1:** Evaluation constructs, outcomes, and data sources.

Construct		Outcomes
**Aim 1: implementation**		
	Reach: is the IPV^a^ screening expansion reaching its intended target (all veterans)?	Proportion of veterans screened out of those eligible for screening and representativeness of screening within the target population^b^ Differences and disparities between those who were and were not screened across patient characteristics (eg, gender, age, race, ethnicity, sexual orientation, and marital status)^b^
	Effectiveness: is the RHS 3.0^c^ screening expansion effective?	Increased frequency of IPV detected among veterans^b^ Increased rate of referrals following screen positives^b^ Clinicians’ and veterans’ experiences with and perceptions of the RHS 3.0^d^ Unintended consequences of the RHS 3.0 rollout^d^
	Adoption: what is the uptake of screening across VHA^e^ facilities and clinical setting?	Proportion of VHA facilities using the RHS 3.0^b^ Proportion of screenings completed using the RHS 3.0 across clinical settings^b^ Barriers and facilitators related to adoption of the RHS 3.0, guided by the CFIR^d,f^
	Implementation fidelity: to what extent is the RHS 3.0 being implemented as intended?	Degree of fidelity (ie, implementation per protocol)^b^ Contextual factors related to fidelity, guided by the CFIR^f^
	Maintenance: is the RHS 3.0 expansion being sustained over time?	Proportion of veterans screened overall and across patient subgroups (eg, gender) using the RHS 3.0 at years 2 and 3^b^ Percent of VHA facilities using the RHS 3.0 at years 2 and 3^b^ Number of unique clinicians using the RHS 3.0 at years 2 and 3^b^
**Aim 2: impact**		
	Connection to and use of health care	Health care use following positive screening; same day visits as a result of screening^b^ Engagement and retention in health care services following positive screens^d^ Sense of support and connection to VHA as a result of screening^d^ Satisfaction with the IPV screening encounter^d^
	Connection to resources	Use of VHA resources for social services or essential needs, such as housing, financial support, food resources, transportation, immigration services, legal aid, and law enforcement assistance (eg, restraining orders)^b^
	Other clinical and health outcomes	Identification and follow-up of high-risk IPV cases (ie, danger of lethality or serious injury)^b^ Number of safety plans completedb Clinical decisions made or actions taken as a result of screeningd

^a^IPV: intimate partner violence.

^b^Indicates electronic health record data extracted from the corporate data warehouse.

^c^RHS 3.0: Relationship Health and Safety Clinical Reminder version 3.0.

^d^Indicates data resulting from qualitative interviews.

^e^VHA: Veterans Health Administration.

^f^CFIR: Consolidated Framework for Implementation Research.

### Ethical Considerations

This evaluation is a quality improvement (QI) initiative jointly supported by the VA Care Management and Social Work Service’s IPV Assistance Program; the VA Quality Enhancement Research Initiative (QUERI); and the IPV Center for Implementation, Research, and Evaluation (IPV-CIRE) at VA Connecticut Health care System. The PRISM Initiative was designed for internal purposes in support of VA QI as an internal operations evaluation designated as nonresearch by VA, thus not requiring institutional review board approval [42]. Empirical research conducted with data collected from this QI initiative was approved by the VA Connecticut Health Care System institutional review board (protocol #1792152).

### Quantitative Procedures and Analyses

Quantitative data grounded in the RE-AIM outcomes will be extracted from the CDW and analyzed using a longitudinal observational design with repeated measurement periods at baseline (T0), year 1 (T1), and year 2 (T2) [38]. We operationalized reach as the proportion and representativeness of veteran patients who were administered IPV screening, and those administered the RHS 3.0 specifically, calculated as veterans screened out of those eligible for screening. Eligibility for screening was defined as having at least 1 VHA health care encounter during the observation period. To examine representativeness and potential disparities between those screened and not screened, we will use generalized linear mixed models (GLMM) to assess differences by patient characteristics (gender, age, race, ethnicity, sexual orientation, marital status, rurality, and housing instability status) over each of the 3 time points while specifying facilities as random effects to account for nesting [43,44]. We will also examine the change in the probability of being screened over time through GLMMs specifying facilities and patients as random effects. Additionally, we will examine whether the effect of time varies significantly across facilities. In post hoc analyses, we will investigate whether the change in the probability of being screened over time differs by patient characteristics.

Effectiveness, for this evaluation, focuses on determining the extent to which the RHS 3.0 expansion itself is effective. We defined the effectiveness of the expansion as whether there is an increase in the proportion of IPV cases detected, referrals offered, and universal education provided following the implementation of the RHS 3.0. Using the quantitative CDW data, we will calculate changes in the proportion of these factors following positive screens through the same GLMM process described above. Effectiveness will also be examined through the exploration of qualitative data regarding clinicians’ and veterans’ experiences with and perceptions of the RHS 3.0 and its potential unintended consequences. Adoption was operationalized as differences in screening uptake by facility and clinical setting [38]. We will combine stop codes, identifiers used by the VHA to track which clinic group and location provided a service, into meaningful categories of services (eg, primary care mental health, social work, etc) to examine descriptive statistics (frequencies and range) of screening uptake across medical center facilities and clinical settings. We will also assess adoption through an exploration of the barriers and facilitators related to IPV screening expansion via qualitative data.

Implementation fidelity was defined as the extent to which IPV screening and response procedures are implemented as intended (ie, per IPV Assistance Program guidelines) [20,38]. For example, we will identify the percentage of veterans screened out of those eligible, completion of the secondary screen based on positive primary screen responses, referrals offered, and universal IPV education provided. We will also explore contextual factors related to fidelity reported during qualitative interviews. Finally, to examine maintenance of the RHS 3.0 expansion, we will identify whether the proportion of veterans screened (and proportion screened across patient subgroups), percent of facilities using the RHS 3.0, and number of unique clinicians using the RHS 3.0 remains at, above, or below T0 levels during T1 and T2 [38].

In addition to examining the implementation of the IPV screening expansion, the PRISM initiative will evaluate the impact of expanded IPV screening on patients, clinicians, and the health care system as a whole. Unique from RE-AIM’s effectiveness domain, which targets whether the screening expansion itself is effective, determining the impact of expanded screening will involve examining linkages between screening and connection and engagement to VHA services and resources. Leveraging available CDW data, we will examine patients’ connection to, and use of, health care and social support services following IPV screening, including services for physical and mental health conditions and resources for social services or essential needs (see Table 1 for detailed description). Examination of CDW data will also enable the identification of responses to IPV cases identified as high risk for lethality or serious injury, including a number of safety plans completed or same-day consults placed. Additional analyses will include a calculation of the frequency of services received in the 60 days following a positive screen and the same day. We will determine associations between positive screens and health care or resource use by using GLMMs with facilities as random effects, controlling for service use in the 60 days prior to the screen. We will also examine rates of high-risk cases detected from secondary screener results and the proportion of safety plans completed among high-risk cases.

### Qualitative Procedures and Analyses

#### Guiding Frameworks

We will use the CFIR 2.0 [39] as an organizational and explanatory determinants framework to (1) categorize multilevel factors critical to RHS 3.0 implementation across VHA facilities and clinical settings, (2) identify potential barriers and facilitators of implementation, and (3) examine the impact of screening on patients. The CFIR 2.0 includes 49 constructs across 5 overarching domains that have been shown to influence program implementation, and they are (1) innovation, (2) inner setting, (3) outer setting, (4) individuals, and (5) implementation process [39]. Following guidance to use constructs most salient for particular initiatives under study [45], we selected the most relevant constructs from the CFIR 2.0 and CFIR Outcomes Addendum [46] to guide the development of interview guides with veterans and clinicians and analyses of qualitative data for this evaluation. See Table 2 for a full list of CFIR constructs guiding qualitative data collection and analysis.

**Table 2:** CFIR^a^ domains and constructs for evaluation of the RHS 3.0^b^ expansion.

Level and construct			C^c,d^ or P^d,e^
**Innovation characteristics**			
	Evidence strength and quality	C	
	Adaptability	C or P	
	Complexity	C or P	
**Outer setting**			
	External policies and incentives	C	
	Patient needs and resources	C or P	
**Inner setting**			
	Culture and climate	C	
	Compatibility	C	
	Relative priority	C	
	Access to information	C	
**Individuals: recipients (P) and deliverers (C)**			
	Self-efficacy	C	
	Need and appropriateness	C or P	
	Capability, opportunity, and motivation	C	
	Acceptability	C or P	
	Feasibility	C	
	Impact and outcomes	C or P	
**Process**			
	Tailoring strategies	C	
	Adapting	C	

^a^CFIR: Consolidated Framework for Implementation Research.

^b^RHS 3.0: Relationship Health and Safety Clinical Reminder version 3.0.

^c^C: clinicians.

^d^Indicating which constructs will be reflected in corresponding interview guides.

^e^P: patients.

#### Patients

Leveraging EHR data, we will use purposeful sampling [47] to identify veteran patients who screened positive for IPV and created random stratified samples by gender and ensure patient presentation across geographical locations and clinical settings. We will generate batches of eligible veterans monthly to ensure that veterans are interviewed within 2 to 4 months of their screening encounter and have sufficient memory of the experience. We will send recruitment letters to those potentially eligible informing them of the study and inviting them to contact the study team to opt in or out of participation. Veterans with cognitive, language, or other impairments that prevent full participation in the study will not be eligible to participate. To ensure diversity in perspectives across interviews, we aim to include veterans from varying demographic backgrounds and experiences. We will encourage this diverse makeup by ensuring that our sample includes at least 20 women and 20 men, 25% racial or ethnic minority veterans, and 25% veterans aged 55 or older; we will also seek representation from each region of the country [48,49]. Recruitment will continue until we reach thematic saturation [50]. Interviews will be approximately 45 minutes and veterans will be compensated US $50 for their participation. Veterans’ participation will be voluntary and their responses to interview questions will not be shared with any clinician providing them VHA services. Interview data will be presented using deidentified representative quotes and in aggregate through the development of qualitative themes.

#### Clinicians

We will leverage the EHR data to identify clinicians who used the RHS 3.0 to screen patients of all genders for IPV to create a roster of potentially eligible clinicians. Using this roster, we will ensure clinician representation across clinician discipline, geographical location, and clinical settings. We will reach out to potentially eligible clinicians via email informing them of the study, confirming that they have conducted at least 5 screenings with women and at least 5 with men, and inviting them to participate in a qualitative interview about their experiences and perceptions. We aim to include at least 25 clinicians, although our final sample will be determined by thematic saturation [50]. Interviews will be 45-60 minutes in length.

#### National IPV Assistance Program Leaders

Using an established ethnographically informed method of guided discussion [51], we will conduct biannual 30-60 minute interviews with 3-4 key national IPV program leaders involved in supporting the RHS 3.0 expansion and program office partners, at the leaders’ request. These discussions will enable us to systematically document and understand the implementation plan and process, as well as provide opportunities for the national implementers to engage in “periodic reflections” with our team [51]. Discussion notes will be coded to reflect key CFIR domains of interest and emergent themes, which will be analyzed in triangulation with our other qualitative data sources (ie, clinician interviews) and quantitative data (ie, CDW-based RE-AIM domains).

#### Qualitative Data Analysis

We will use a hybrid deductive-inductive thematic analysis approach [52] to analyze the qualitative data, with initial codes informed by specified CFIR constructs (Table 2). Interview transcripts will be coded and summarized, then consolidated into matrices by CFIR constructs. Multiple team members will conduct data analyses. Using a rigorous, team-based approach, we will complete the following steps: (1) develop a start list of codes based on the CFIR constructs and interview guides (to which we will add emergent codes); (2) code the transcripts; (3) transpose and systematize data into summary templates; (4) organize the data into matrices to note trends, similarities, and differences; and (5) synthesize into findings. As new codes arise, earlier transcripts will be recoded [53]. This iterative process will continue until all themes have been identified. Discrepancies will be resolved through consensus discussions [54]. Qualitative findings will support the planned evaluation by (1) describing conditions necessary for clinicians to effectively screen all veterans across health care settings and barriers to doing so; (2) identifying contextual factors that will inform future implementation strategies needed to enhance the expansion of the RHS 3.0; and (3) revealing veterans’ experiences with, and outcomes related to, disclosing IPV during screening encounters, including the impact of screening on their health care and service use, satisfaction with the screening and response encounter, and sense of connection with VHA and clinicians.

#### Patient Engagement

This evaluation will include a Veteran Engagement Board (VEB) to accurately represent the complex and diverse experiences of veterans and ensure that our findings are meaningful and accessible to the patient population we are striving to serve [55]. Through the VEB, we will include veterans’ voices and perspectives in each phase of the evaluation and aim to increase shared decision-making across a diversity of perspectives [56]. Although we will gain important knowledge through qualitative interviews with veterans to understand the impact of the RHS 3.0 screening and response protocol expansion on patients, involving veterans in research to understand their experiences differs meaningfully from engaging them in the research process to ensure that all phases of the work are veteran-centric and guided and informed by veteran perspectives. The VEB will actively inform the PRISM initiative through regularly occurring meetings and discussions focused on activities across all stages of the project (ie, planning, data collection, interpretation of results, and dissemination of findings).

## Results

The PRISM initiative was funded in October 2023. We have developed the qualitative interview guides, obtained institutional review board approval, extracted quantitative data for baseline analyses, and began recruitment for qualitative interviews. Quantitative analyses will take place in 2024 (T0), 2025 (T1), and 2026 (T2). Qualitative interviews and analyses will take place between April 2024 and October 2025. Reports of progress and results will be made available to evaluation partners and funders through quarterly and end-of-year reports. Evaluation findings will also be intermittently disseminated through peer-reviewed journals and presentations at scientific meetings. All data collection and analyses across time points are expected to be completed in June 2026.

## Discussion

This study protocol outlines a mixed methods evaluation of IPV screening expansion in the VHA, conducted in partnership with the VA National IPV Assistance Program. Findings generated from the evaluation of the IPV screening expansion will provide a comprehensive understanding of the reach, effectiveness, adoption, implementation, maintenance, and impact of the expanded IPV screening and response protocol, the RHS 3.0. Additionally, findings will determine the scope of IPV detected in the VA patient population through screening during routine clinical health encounters, knowledge essential to inform clinical practice and policy. These data will generate knowledge regarding IPV disclosures among subgroups of veterans previously not targeted for IPV screening (eg, men, women above reproductive age, and transgender and nonbinary veterans) and those from underserved or vulnerable populations, disparities in IPV screening and outcomes, and patient subgroups at heightened risk for IPV and IPV-related injury and lethality. Little research has examined IPV screening and referral outcomes within these populations, specifically among men and transgender or nonbinary patients (who are at increased risk of IPV compared to cisgender patients [13-15]). Because the majority of IPV screening initiatives to date have targeted women, this work will expand the field’s knowledge regarding other subgroups’ experiences and perceptions of screening and their willingness to disclose IPV during health care encounters.

One limitation of this national evaluation initiative is our ability to access data across all VA sites. The VA is currently undergoing an EHR modernization, including transitioning to a new EHR system [57]. These changes may impede our ability to identify and extract necessary data at VA sites that have transitioned to a new EHR system. Additionally, we are limited by our evaluation method. Although a staggered implementation rollout or use of control group sites may enhance evaluation by allowing for comparisons of strategies, the national IPV program partners are prioritizing a full-scale national rollout, currently underway, limiting the possibility of a staggered approach.

This initiative also has critical policy and clinical practice implications. Through the course of this project, we will develop essential evaluation tools for monitoring and improving screening implementation in the VHA over time. The VA’s Office of the Inspector General identified the need for systematic and high-quality tracking of IPV-related programmatic outcomes data, particularly regarding IPV screening implementation outcomes [58], an area that this project will directly address. This evaluation will provide critically needed systems and clinical data to VHA policy leaders to inform national programming and enable tracking over time. Evaluation of the IPV screening expansion will result in recommendations for future IPV screening implementation initiatives and adaptations, including potential comparisons of implementation strategies for future studies leading to the optimization of IPV screening implementation across the health care system.

## Abbreviations

CDW: corporate data warehouse
CFIR 2.0: Consolidated Framework for Implementation Research
EHR: electronic health record
GLMM: generalized linear mixed models
IPV: intimate partner violence
IPV-CIRE: intimate partner violence Center for Implementation, Research, and Evaluation
PRISM: Partnered Evaluation of Relationship Health Innovations and Services through Mixed Methods
QI: quality improvement
QUERI: Quality Enhancement Research Initiative
RE-AIM: Reach, Effectiveness, Adoption, Implementation, and Maintenance
RHS 3.0: Relationship Health and Safety Clinical Reminder version 3
VA: Department of Veterans Affairs
VEB: Veteran Engagement Board
VHA: Veterans Health Administration

## Data Availability

The data sets generated and analyzed during this study are not publicly available due to requirements of compliance with government procedures. Those interested in deidentified data underlying this initiative can send an email request to the corresponding author.
